# Establishing Functional Relationships between Abiotic Environment, Macrophyte Coverage, Resource Gradients and the Distribution of *Mytilus trossulus* in a Brackish Non-Tidal Environment

**DOI:** 10.1371/journal.pone.0136949

**Published:** 2015-08-28

**Authors:** Jonne Kotta, Katarina Oganjan, Velda Lauringson, Merli Pärnoja, Ants Kaasik, Liisa Rohtla, Ilmar Kotta, Helen Orav-Kotta

**Affiliations:** 1 University of Tartu, Estonian Marine Institute, Department of Marine Biology, Mäealuse 14, 12618 Tallinn, Estonia; 2 University of Tartu, Institute of Ecology and Earth Sciences, Chair of Zoology, Vanemuise 46, 51014, Tartu, Estonia; 3 Nova Southeastern University, Halmos College of Natural Sciences and Oceanography, 8000 North Ocean Drive, Dania Beach, Florida, United States of America; GEOMAR Helmholtz Centre for Ocean Research Kiel, GERMANY

## Abstract

Benthic suspension feeding mussels are an important functional guild in coastal and estuarine ecosystems. To date we lack information on how various environmental gradients and biotic interactions separately and interactively shape the distribution patterns of mussels in non-tidal environments. Opposing to tidal environments, mussels inhabit solely subtidal zone in non-tidal waterbodies and, thereby, driving factors for mussel populations are expected to differ from the tidal areas. In the present study, we used the boosted regression tree modelling (BRT), an ensemble method for statistical techniques and machine learning, in order to explain the distribution and biomass of the suspension feeding mussel *Mytilus trossulus* in the non-tidal Baltic Sea. BRT models suggested that (1) distribution patterns of *M*. *trossulus* are largely driven by separate effects of direct environmental gradients and partly by interactive effects of resource gradients with direct environmental gradients. (2) Within its suitable habitat range, however, resource gradients had an important role in shaping the biomass distribution of *M*. *trossulus*. (3) Contrary to tidal areas, mussels were not competitively superior over macrophytes with patterns indicating either facilitative interactions between mussels and macrophytes or co-variance due to common stressor. To conclude, direct environmental gradients seem to define the distribution pattern of *M*. *trossulus*, and within the favourable distribution range, resource gradients in interaction with direct environmental gradients are expected to set the biomass level of mussels.

## Introduction

A key mission in ecology is to understand biotic patterns and their changes in nature. In order to achieve such an understanding in the marine realm, ecologists have initiated a multitude of projects aiming to map marine biota or performed experiments to demonstrate interactions between physical environment and organisms. However, as direct mapping of biota is extremely costly in marine environment, modelling has become an unavoidable tool, and several refined statistical approaches have been already applied in the field [1]

Distribution patterns of species depend on their ecological niche, which consists of a multi-dimensional environmental space. In general, non-independent effects are common in nature [2,3] and, therefore, neither the species niche nor the resulting distribution range can be predicted from separate effects of an individual environmental variable. A suitable habitat is often defined by complex interrelationships among a multitude of environmental variables that can be largely divided into three broad categories [4]. These include indirect environmental gradients, resource gradients and direct environmental gradients. Indirect environmental gradients can often be easily measured, but represent only proxies for a set of underlying gradients, which affect organisms directly while it may be difficult to measure or disentangle the effects of these underlying gradients [5,6]. Water depth can be viewed as a typical indirect environmental gradient in the marine realm. Resource gradients are substances being consumed and direct environmental gradients represent features that have direct physiological impact on growth but are not consumed. The picture gets more complicated as the same factor may have an impact simultaneously via different pathways. For example, water movement can indirectly affect the habitat of suspension feeding bivalves by modifying sedimentation rates or affect sessile mussels directly by physically disturbing or detaching animals [1,7]. Furthermore, the benthic suspension feeding mode and sedentary lifestyle of mussels prescribe an intrinsic need for a vector of food delivery. Thereby, water movement can impact benthic suspension feeders also through a third pathway, by modifying the resource supply while limiting the amount of food reached by mussels [8,9].

Although the niche concept introduced resource axes and the importance of competition [10], most of the ecological niche modeling has been dealing with abiotic factors only, without considering interspecific interactions and resources [11–13]. Therefore, it is largely unknown how biotic environment interacts with Hutchinsonian fundamental niche space in structuring real communities [14]. The realized niche of a species, however, depends largely on biotic interactions with other species [15–17]. Thereby, here we distinguish besides direct/ indirect environmental and resource gradients also the fourth type of gradients, namely the abundances of ambient species or organism groups other than direct resources, but either competitors, predators or facilitators. We refer to this gradient type as biotic interaction gradients.

Benthic suspension feeding mussels are an important functional guild in coastal and estuarine ecosystems. This functional guild feeds on suspended food, usually microalgae, from bottom-reaching water masses [18]. Despite a large body of field and experimental works [19,20] we still lack knowledge on how various environmental gradients separately and interactively shape the distribution patterns of suspension feeding mussels in different ecosystems. The reason for this is, firstly, because the distribution of suspension feeders is controlled by a large number of processes involving both benthic and pelagic environments (e.g. substrate colonization, water movement, phytoplankton production, physical disturbances) as well as many interactions between these processes [1,21]. Secondly, due to this complexity in driving forces, the direction and magnitude of environmental impact on mussels is expected to vary highly among different ecosystems [22,23]. To date, the distribution patterns of mussels have been extensively studied in tidal habitats [19,24–26] whereas studies on nontidal areas are still scarce. Contrasting to tidal areas, mussels inhabit solely subtidal zone in nontidal waterbodies [27] and driving factors for mussel populations are expected to differ from the tidal areas [23]. In the tidal zone species are constantly challenged by fluctuating environmental conditions and the biotic patterns often reflect the stress tolerance of species [28–30]. On the other hand, subtidal areas offer species some stability; thus, the distribution patterns of mussels are expected to be shaped primarily by habitat and food availability as well as predation pressure [31].

The rising interest in marine habitat mapping has resulted in numerous modelling studies focussed on the distribution of species [32–35]. However, traditional statistical modelling may not be the most rewarding way to understand environmental-species relationships, as it starts by assuming an appropriate data model, and model parameters are then estimated from the data [36]. Due to the lack of a solid knowledge on how the external environment impacts the species that we are trying to model, the predictive performance of these models is expected to be moderate. On the other hand, due to time constraints and limited man power, experimental studies cannot resolve causal connections beyond one or two environmental variables. Moreover, experiments are only seldom replicated in space and time. As a consequence, the experimental approach can provide us a very localized snapshot, but not a generic understanding on environment-biota relationships.

Machine learning provides a theoretical framework that moves beyond traditional paradigm boundaries. Considering „complex realism”and our weak theoretical foundations, modelling is seen here as a sophisticated tool to improve our understanding on the relationship between environment and biota. By contrast to traditional methods, machine learning avoids starting with a data model and rather uses an algorithm to learn the relationship between the response and its predictors [37]. But even here some ecological understanding is a prerequisite when it comes to selecting environmental variables for the model. Specifically, in order to succeed in identifying and quantifying relationships between the environment and biota, the model should incorporate at least the most important direct and resource gradients as well as recapture multitude of interactions between these environmental gradients and biota. The novel predictive modelling technique called Boosted Regression Trees (BRT) combines the strengths of machine learning and statistical modelling. BRT has no need for prior data transformation or elimination of outliers and can fit complex nonlinear relationships. The BRT also avoids overfitting the data, thereby providing robust estimates. What is the most important in the ecological perspective: it automatically detects and models interactive effects between predictors. The method copes with different non-linear relationships including thresholds and unimodal responses which are common in ecological data but difficult to analyse using more traditional methods. Due to its strong predictive performance, BRT is increasingly used in ecology [38,39]. Although, we admit that the results of distribution modelling are purely correlative and causal interpretations need to be validated by future experimental manipulations, machine learning algorithms enable a powerful initial insight to the key drivers as well as to the interactions between the environment and the biota.

Blue mussels consist of a group of three closely related taxa, known as the *Mytilus edulis* complex. The common mussel *Mytilus edulis* in sensu stricto is native to the North Atlantic, the Mediterranean mussel *Mytilus galloprovincialis* is native in the Mediterranean, the Black Sea and Western Europe and the bay mussel *Mytilus trossulus* is native to North Pacific, northern parts of the North Atlantic and Baltic Sea. The taxa can hybridise with each other, if present at the same locality. *M*. *trossulus* inhabits both subtidal as well as intertidal areas, tolerates a wide range of environmental conditions and therefore gains high biomasses at different habitat types [40]. This makes the species a good model organism to improve our understanding on the roles of multiple environmental gradients on the distribution of benthic suspension feeders. In the brackish nontidal Baltic Sea, *M*. *trossulus* is an important organism in various hard and mixed bottom subtidal habitats. Here *M*. *trossulus* coexists with *M*. *edulis*, but as a key ecological differentiation *M*. *trossulus* tolerates lower salinity compared to *M*. *edulis* and thereby distributes almost the whole range of the Baltic Sea [41,42]. However, there are no pure *M*. *trossulus* in the Baltic Sea with all mytilids being hybrids, with varying fractions of *M*. *edulis* alleles in their genomes [43].

In the present study, we aimed to describe the realized niche of the mussel *M*. *trossulus* in the northeastern Baltic Sea, both in terms of distribution and the size of populations. We used the BRT modelling (1) to quantify the relative contribution of resource, abiotic environmental and biotic interaction gradients on the distribution of *M*. *trossulus* in the Baltic Sea (2) We also sought how the availability of resources affects the standing stock of species and (3) how biotic interactions and different direct environmental gradients including key disturbances either separately or interactively modulate the resource-biomass relationship.

We expected that at the regional scale, salinity is considered as the main factor driving the distribution of *Mytilus trossulus* [42]. Locally, however, a large array of environmental variables such as substrate type, water temperature, flow velocity, winter-time ice scour, are expected to either separately or interactively shape the distribution pattern of mussels [7,44–47]. We also expected that within a favourable habitat, the availability of food resources defines the biomass of species [47]. Nevertheless, resource gradients in this space may interact with direct environmental gradients, which act as valves regulating the availability of resources. As benthic suspension feeders link two spatially distinct systems, specific abiotic environmental conditions may be of utmost importance for them in determining the amount of resource to be received [47–49]. In addition, disturbance may reduce or ultimately even disrupt the link between resource parameters and the distribution of species [50]. This may explain why some highly trophic areas with e.g. sufficient amount of hard bottom and suitable salinity lack dense mussel populations [51]. Finally, we expect that the interspecific competition between mussels and other biota is moderate, rarely outperforming the effects of abiotic environmental disturbances [44]. It is expected that macroalgae compete with mussels for substrate, although, this has not been experimentally demonstrated in the Baltic Sea. Instead, mussels are known to facilitate the growth of macroalgae [52] and, thus, mutualistic interactions between mussels and macroalgae (e.g. dampening different types of disturbances, intensifying turbulent flows at the bottom-water interface) may actually outweigh a potential reduction in advection by canopy macroalgae [53]. As compared to the oceanic waters, the Baltic Sea lacks the major epibenthic predators and therefore the predation pressure on mussels is also low [27,54]. Predation by vertebrates in the study area is rare, declining and hardly detectable, therefore, we decided not to include predation to the distribution model [55].

## Methods

### 1. Study area

**Fig 1:**
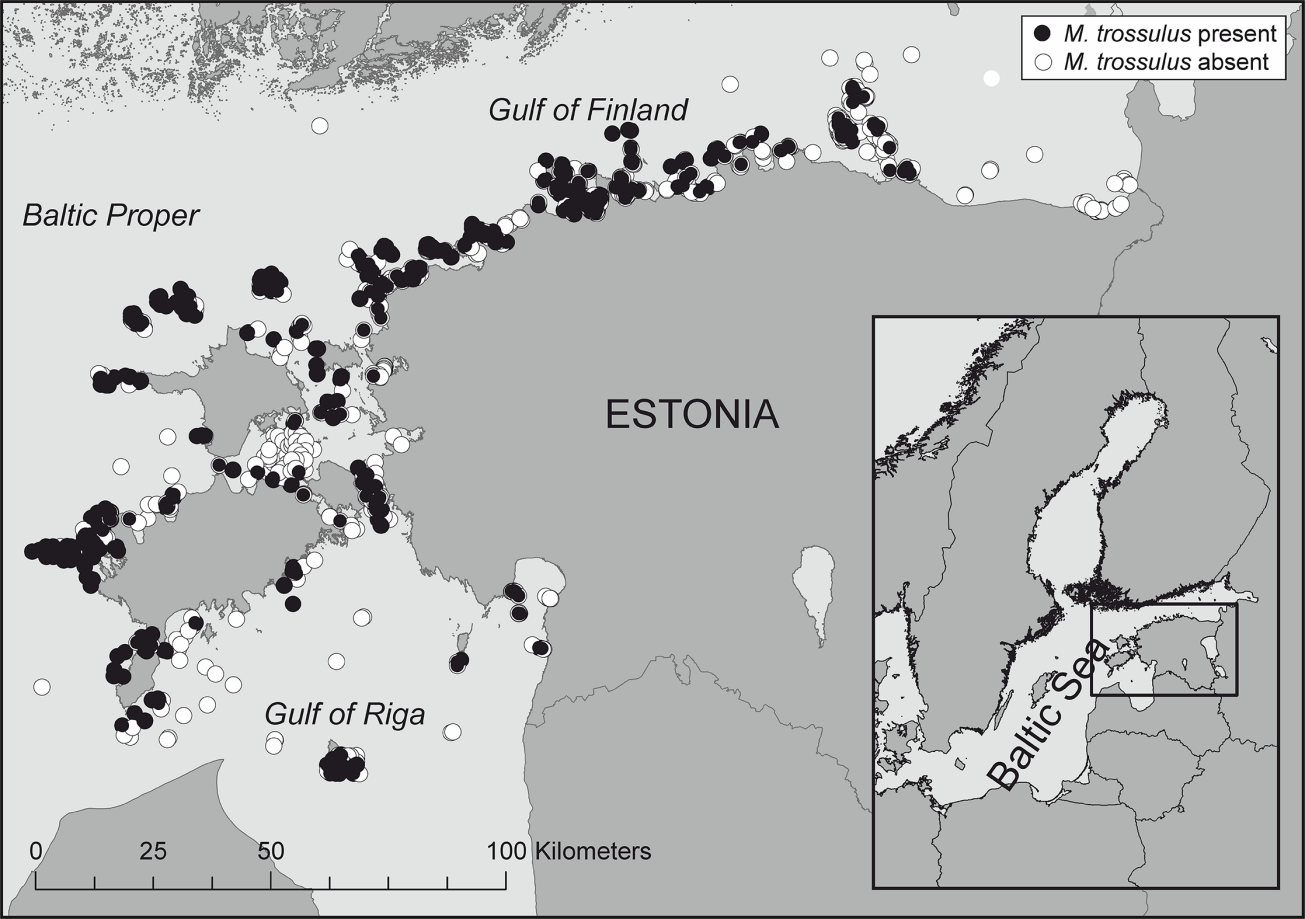
Map of the sampling stations in the study area. Filled circles indicate the locations of *M*. *trossulus*.

The study area lies in the northeastern Baltic Sea, in the Estonian coastal sea (Fig 1). It is characterized by fully submerged habitat due to the absence of tides, although very shallow waters may be irregularly exposed by the action of wind. Salinity is constantly low and close to the physiological tolerance of mussels. Opposing to more saline range of the species, invertebrate predation is absent in the study area [27,54]. The study area encompasses major geomorphological structures including different types of soft, limestone and granite bedrock, allowing thus to generalize the obtained results over large parts of the Baltic Sea [56,57]. Large parts of the study area are relatively flat and shallow, lacking steep slopes. Shallow areas may also be subjected to intense winter-time ice scour. Wave energy is lower than on the coasts of large oceans, but may still be remarkable for the bottom fauna at shallow exposed sites, especially during autumn and winter storms. Some areas are subjected to local upwelling events induced by wind conditions. Often, angiosperm or macroalgal communities inhabit these bottoms at depths down to 20 m. The mussel *M*. *trossulus* exhibits generally low biomass and sparse distribution. Only at very exposed open-sea areas, the biomass may exceed 1 kg dw m^-2^ [58].

### 2. Biological data

Altogether 3585 stations were sampled within the Estonian territorial waters during the ice-free seasons between 2005 and 2009. The majority of stations were sampled only once. Within each waterbody approximately 15 stations were sampled annually. In order to establish the sampling stations, a grid of rectangular cells was generated with cell sizes of 300 m using the Spatial Analyst tool of ArcInfo 10 [59]. Then we calculated the values of wave exposure and inclination of coastal slopes for each grid cell (see below). The exposure and slope classes were combined to the available information on depth and bottom sediments (divided into clay, silt, sand, gravel, boulder and rock bottoms) available in the databases of the Estonian Marine Institute. Sampling sites were located randomly in a way that each combination of exposure, slope, depth and sediment class had a comparable number of sampling sites (Table 1).

**Table 1:** Measured environmental variables in the overall sampling area and in the area where *M*. *trossulus* was found.

Variable	Unit	Sampling area			Distribution area		
		Mean	Min	Max	Mean	Min	Max
Depth	m	11.77	0.10	75	8.87	0.2	47
Exposure	m^2^ s^-1^	229020	5672	968957	277950	5672	968957
Slope	°	0.66	0	13.47	0.79	0	10.56
Ice thickness	m	0.28	0	0.50	0.26	0	0.48
Temperature	°C	12.95	0.03	22.23	12.88	0.03	22.23
Salinity	psu	6.26	3.70	8.05	6.66	4.42	7.93
Oxygen	mmol m^-3^	319	0	376	325	0	375
Velocity	cm s^-1^	3.75	0	15.26	3.58	0	13.34
Silt clay cover	%	13.34	0	100	6.22	0	100
Sand cover	%	38.12	0	100	21.96	0	100
Boulder cover	%	37.87	0	100	58.15	0	100
Chlorophyll *a*	mg m^-3^	19.54	0.66	45	19.00	0.66	45
Plant cover	%	31.65	0	100	44.43	0	100

At each sampling site the coverage of different sediment types (rock, boulders, pebbles, gravel, sand, silt) and macrophytes (both macroalgae and higher order vegetation) was estimated either directly by diver or remote underwater video device. The underwater camera was set at an angle of 35° below horizon to maximise the field of view and the range of the forward view was about 2 m in clear waters.

In addition at each sampling site quantitative samples of *M*. *trossulus* were collected in three replicates either by a diver using a standard bottom frame (0.04 m^2^) on hard bottoms, or by a quantitative Ekman-Lenz grab sampler (0.02 m^2^) on soft bottoms. Although the samples were collected using two different methods with different accuracy, these two methods are comparable in the case of *M*. *trossulus* with limited escape abilities and relatively homogenous seafloor area. Samples were sieved at the field on 0.25 mm mesh screens. The residues were stored at −20°C and subsequent sorting and counting of species was performed in the laboratory using a stereomicroscope. The dry weight of mussels was obtained after drying the individuals with shells at 60°C for 2 weeks.

Biomass sampling and analysis followed the guidelines developed for the HELCOM COMBINE programme [60]. According to the Protection Rules of the Estonian coastal waters, biological sampling does not require specific permits or approvals. The study area is not privately-owned and the study did not involve endangered or protected species.

### 3. Environmental data

A set of environmental variables were chosen for the analyses based on the theoretical assumptions of the role of environment on the mussel distribution (Table 1). The values of water temperature, salinity and water velocity were obtained from the results of hydrodynamical model calculations from 2005−2009. As annual averages, minima and maxima of the studied hydrological variables were highly intercorrelated, we used annual averages in the final models. The calculations were based on the COHERENS model which is a primitive equation ocean circulation model. It was formulated with spherical coordinates on a 1′ × 1′ minute horizontal grid and 30 vertical sigma layers. The model was forced with hourly meteorological fields of 2 m air temperature, wind speed, wind stress vector, cloud cover and relative humidity. The meteorological fields were obtained from an operational atmospheric model. The model was validated against water level, temperature, salinity and water velocity measurements from the study area [61].

Winter-time ice disturbance is the key disturbance for macrophyte and benthic invertebrate communities in the Baltic Sea range [62,63]. Finnish Meteorological Institute provided ice cover over the study area for the investigated period. Ice cover and thickness were produced on daily basis at a nominal resolution of 500 m and were based on the most recent available ice chart and synthetic aperture radar (SAR) image. The ice regions in the ice charts were updated according to a SAR segmentation and new ice parameter values were assigned to each SAR segment based on the SAR backscattering and the ice thickness range at that location.

The amount of available food resources affects the densities of species [64]. For suspension feeders, this could be translated from the amount of organic seston in the water. Water chlorophyll *a* is a good proxy for the food supply of mussels [8]. In this study we used the satellite sensor MODIS Aqua derived water chlorophyll *a* values. This measure is limited to surface waters only; however, due to intensive mixing in our shallow water ecosystem, the satellite derived values represent well near-bottom conditions. Satellite observations were recorded on weekly basis over the whole ice-free period. Cloud, land and other processing flags were identified and masked by NASA Level 2 Ocean Color Processing. The spatial resolution of satellite data was 1 km. Erroneous zero chlorophyll *a* values may occur due to different problems in image processing chain. The erroneous values have to be removed prior to statistical analysis. As in all year round chlorophyll *a* values only very rarely drop below 0.2 in the study area, we used a threshold of 0.1 to filter out all these false zero concentrations.

Another variable affecting mussels along different pathways is exposure to waves [1,7]. Exposure defines the water exchange both between coastal and open sea as well as between water surface and bottom layers [65]. Thus, the interaction between chlorophyll *a* and exposure is expected to indicate the flux of food into the site [50]. Besides being important for resource allocation, wave exposure is also a direct variable transporting larvae and affecting adults directly [45]. The Simplified Wave Model method was used to calculate the wave exposure for mean wind conditions represented by the ten year period between 1 January 1997 and 31 December 2006 [66]. A nested-grid technique was used to take into account long distance effects on the local wave exposure regime. The resulting grids had a resolution of 25 m. In the modelling the shoreline was divided into suitable calculation areas, fetch and wave exposure grids were calculated and subsequently the separate grids were integrated into a seamless description of wave exposure along the study area. This method results in a pattern where the fetch values are smoothed out to the sides, and around island and skerries in a similar way that refraction and diffraction make waves deflect around islands.

Although depth is traditionally regarded amongst the most important parameters describing spatial pattern of mussels [1,48], initially we did not include depth in our model. This is because depth is a surrogate of several direct variables such as light availability, temperature, salinity, pressure, wave action, ice scouring or their combinations [13]. Thus, spatial models that incorporate depth as a independent variable are difficult to interpret due to a multitude of the cause-effect relationships involved. Moreover, it is likely that the depth-biota relationship changes when moving from one geographic region to another, or when extending the study area to include a larger region [1]. However, for environmental management it might be still appealing to find a good approximation of spatial distribution as a function of a single and easy to measure parameter as water depth. Therefore, we run additional model where only depth was used as a predictor of the spatial pattern of mussels.

In order to match temporal patterns relevant to the life span of *M*. *trossulus* and to get rid of potential noise due to the short-term variability of environmental variables, annual averages of hydrophysical variables, wave exposure and water chlorophyll *a* and a wintertime average of ice disturbance were used when modelling the patterns of mussels.

### 4. Boosted Regression Trees (BRT) modelling

Prior to modelling the Pearson correlation analysis between all environmental variables was run in order to avoid situations of including highly correlated variables into the modelling. The correlation analysis showed that most of variables were only weakly intercorrelated at r < 0.1. However, more exposed areas were also characterized by higher salinity (r = 0.60, p < 0.001), lower chlorophyll *a* (r = -0.59, p < 0.001) and lower ice cover (r = -0.44, p < 0.001). In addition, the coverage of stones was inversely related to sand cover (r = -0.59, p < 0.001). Nevertheless, these values are far below a critical threshold when collinearity begins to severely distort model estimation and subsequent prediction [67].

The contribution of different environmental variables on the distribution of *M*. *trossulus* was explored using the Boosted Regression Tree technique (BRT). BRT models are capable of handling different types of predictor variables and their predictive performance is superior to most traditional modelling methods (see e.g. comparisons with GLM, GAM and multivariate adaptive regression splines, [68,69]). While overfitting is often seen as a problem in statistical modelling, this problem can be overcome by using independent data sets. The BRT modelling iteratively develops a large ensemble of small regression trees constructed from random subsets of the data. Each successive tree predicts the residuals from the previous tree to gradually boost the predictive performance of the overall model [38].

The BRT modelling consisted of a two-stage process. In the first BRT model all studied environmental variables (coverage of different sediment fractions, ice thickness, oxygen, salinity, slope, water temperature, wave exposure, velocity, chlorophyll *a*, coverage of macroalgae) were regressed to predict the presence of *M*. *trossulus*. In the second BRT model only the samples containing *M*. *trossulus* were used to predict the biomass of *M*. *trossulus*. In addition, the presence and biomass of *M*. *trossulus* were regressed using only depth as a single independent predictor.

In fitting a BRT the learning rate and the tree complexity must be specified. The learning rate determines the contribution of each successive tree to the final model, as it proceeds through the iterations. The tree complexity fixes whether only main effects (tree complexity = 1) or interactions are also included (tree complexity > 1). Ultimately, the learning rate and tree complexity combined determine the total number of trees in the final model. Following the suggestions by Elith et al. [38] the model learning rate was kept at 0.1 and tree complexity at 5 for both models. It was also checked that the final models had more than 1000 trees. Nevertheless, a selection of model parameters had only marginal impact on model performance with optimal models improving predictions less than 1%. In order to avoid potential problems of overfitting, unimportant variables were dropped using a simplify tool. This tool is a cross-validation based program described by Elith and colleagues [[38], details in Appendix S2]. In order to eliminate non-informative variables, the tool progressively simplifies model, then re-fits the model and sequentially repeats the process until some stopping criterion is reached. Such simplification is most useful for small data sets where redundant predictors may degrade performance by increasing variance. As a consequence, our final models did not include any autocorrelating variables. Model performance was evaluated using the cross validation statistics calculated during model fitting [37]. Thus, when running models a random selection of 80% of the data was used for training the model and the rest of the data i.e. 20% was assigned for testing model accuracy. The BRT modelling was done in the statistical software R using the gbm package [70].

## Results

### 1. Presence of mussels

**Fig 2:**
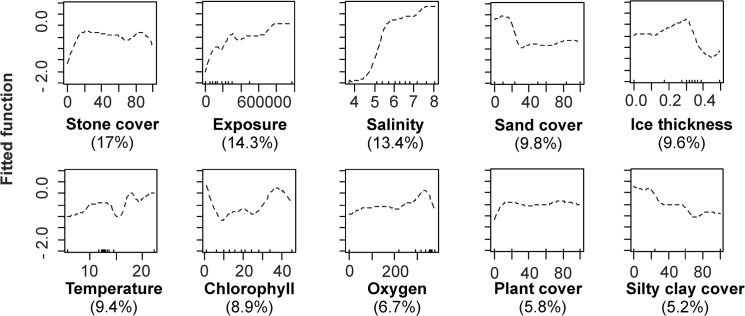
Standardized functional-form relationships showing the effect of environmental variables on the presence of *M*. *trossulus* in the study area, whilst all other variables are held at their means. The variables are ordered by their relative contribution in the BRT model (shown in brackets). Upward tickmarks on x-axis show the frequency of distribution of data along this axis. See the section of methods for further information on environmental variables.

*M*. *trossulus* was found at 1635 stations out of 3585. The BRT modelling with the simplify tool option on described 85% of variability in the presence of *M*. *trossulus*. Altogether ten independent variables were retained in the model. Over 75% of model variability was due to direct abiotic environmental gradients whereas resource gradients (exposure and chlorophyll *a*) contributed less than 25% to the model. In general, direct environmental gradients had strong separate effects while resource gradients impacted the distribution pattern of *M*. *trossulus* either separately or interactively with direct environmental gradients. Functions fitted by BRT models were highly variable in shape, and were mostly non-linear (Fig 2).

The coverage of boulders, exposure to waves, water salinity explained over 50% of the model variability. Other variables contributed much less to the presence of *M*. *trossulus*. The increasing cover of boulders, elevated exposure, salinity as well as moderate ice disturbance separately increased the probability of occurrence of *M*. *trossulus* in the study area. The probability to find *M*. *trossulus* increased with algal cover up to a threshold of 10%. Above this level further increase in algal cover had no effect on mussels. The probability to find *M*. *trossulus* increased both at low and high ends of chlorophyll *a* gradient (Fig 2).

**Fig 3:**
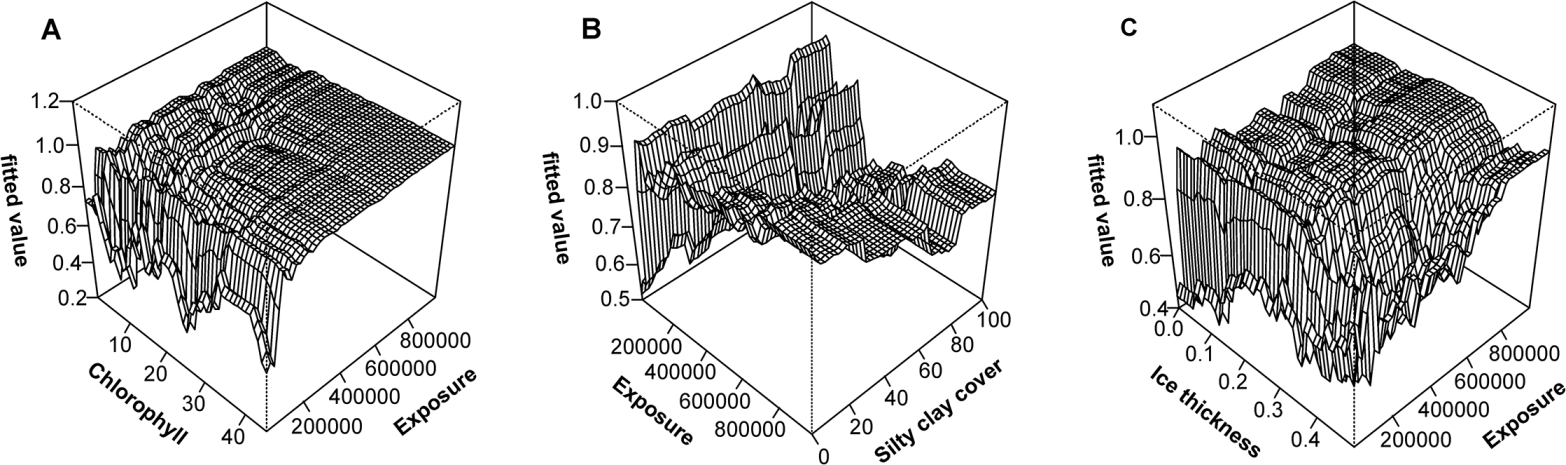
Three-dimensional partial dependence plots in the BRT model for the presence of *M*. *trossulus* in the study area.

Exposure and surface water chlorophyll *a* interactively contributed to the presence of mussels with chlorophyll *a* being important at low exposure values but not at high exposure values. Interestingly, at low exposure chlorophyll *a* value was inversely related to the probability of occurrence of *M*. *trossulus*. In addition, exposure strongly interacted with ice and silt cover. At low ice thickness, the effect of exposure on *M*. *trossulus* was only marginal whereas at high ice thickness elevated exposure exponentially increased the probability of occurrence of *M*. *trossulus*. Similarly, at low exposure the effect of silt on *M*. *trossulus* was moderate whereas at high exposure, elevated silt cover linearly decreased the probability of occurrence of *M*. *trossulus* (Fig 3).

The BRT model including only depth as a single independent predictor explained only 37% of variability in the presence of *M*. *trossulus* in the study area.

### 2. Biomass of mussels

**Fig 4:**
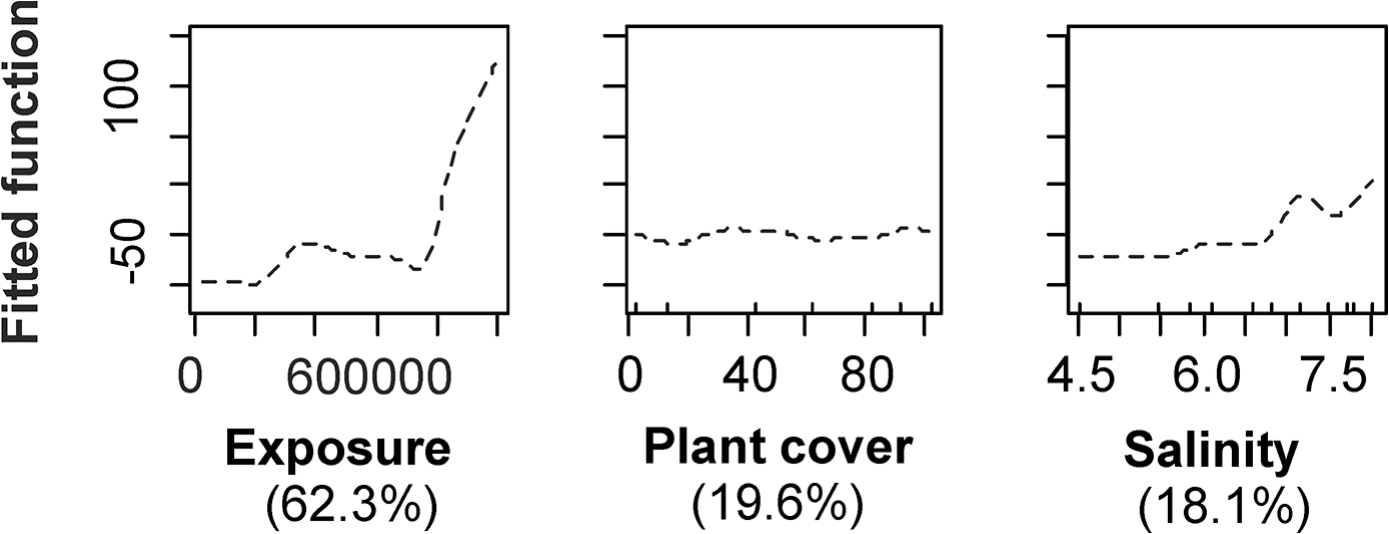
Standardized functional-form relationships showing the effect of environmental variables on the biomass of *M*. *trossulus* within the distribution range of mussels, whilst all other variables are held at their means. The variables are ordered by their relative contribution in the BRT model (shown in brackets). Upward tickmarks on x-axis show the frequency of distribution of data along this axis. See the section of methods for further information on environmental variables.

In areas where mussels were present, the biomass of *M*. *trossulus* was a function of only 3 predictors: exposure, cover of macroalgae and salinity. Nevertheless, the model described only 65% of variability in the biomass of mussels. At low exposure values, the biomass of mussel increased slightly with increasing exposure. Above certain threshold, small increase in exposure resulted in a dramatic increase in the biomass of mussels. Increase in both plant cover and salinity only moderately increased the biomass of mussels. Similar to the presence model, functions fitted by the BRT models were highly variable in shape, and non-linear (Fig 4).

**Fig 5:**
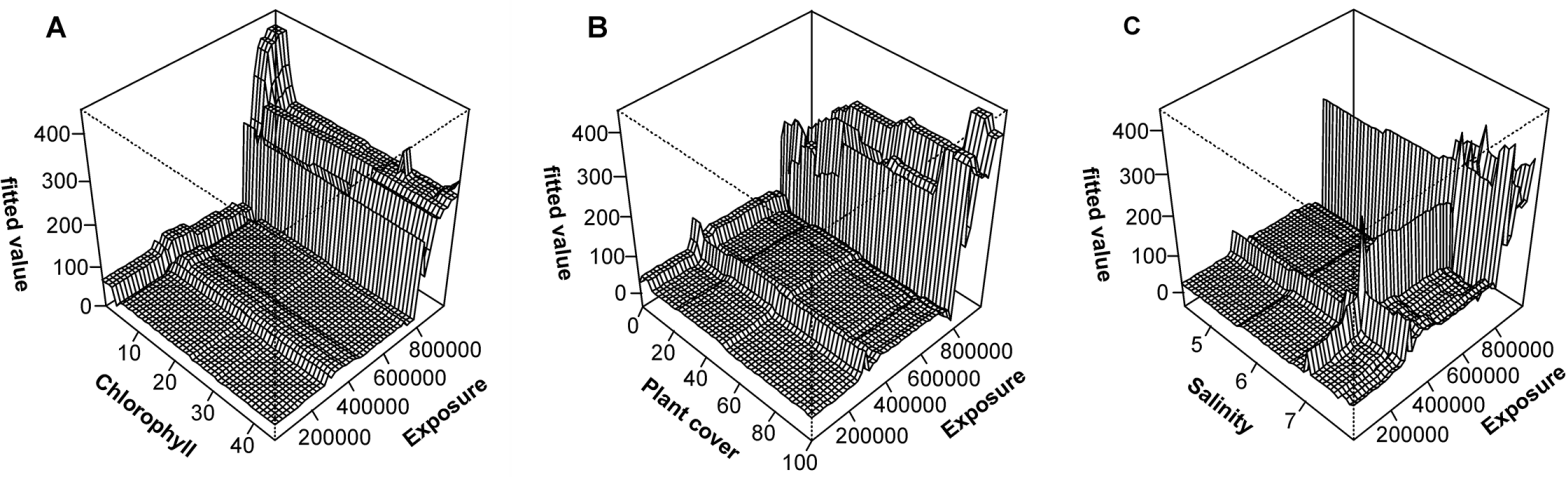
Three-dimensional partial dependence plots in the BRT model for the biomass of *M*. *trossulus* within the distribution range of mussels.

Importantly, exposure and surface water chlorophyll *a* interactively contributed to the biomass of *M*. *trossulus* demonstrating a significant role of resource gradient in the model of mussels’ biomass. High biomasses were found either under conditions of low chlorophyll *a* and high exposure or high chlorophyll *a* and moderate exposure. In addition there were also strong interactions between exposure and the cover of macroalgae and salinity and exposure. At low exposure, relationship between the plant cover and *M*. *trossulus* was weak. At high exposure, however, elevated plant cover was associated to increasing biomass of *M*. *trossulus* with functional relationship indicating two sharp thresholds, each followed by a plateau. Relationship between salinity and *M*. *trossulus* biomass was weak at low exposure whereas increased salinity resulted elevated biomasses at high exposure (Fig 5).

The BRT model including only depth as a single independent predictor explained only 30% of variability in the biomass of *M*. *trossulus* in the study area.

## Discussion

The generic results of our study are that direct environmental gradients seem to define the distribution pattern of the suspension feeding bivalve *M*. *trossulus* and within the favourable distribution range, the resource gradient is expected to have an important role in shaping the biomass distribution of *M*. *trossulus*. As seen from our study, the effects of environmental gradients on biota are complex with a plethora of abiotic and biotic factors simultaneously acting on individual species. Despite of this complexity, the novel machine learning framework offers interpretable description of the multidimensional niche of the species, presented as interrelated relationship curves. Nevertheless, it should be kept in mind that this result is only an imperfect projection of reality, which depends on the selection and number of gradients analysed while simultaneously having all the shortcomings of the input data. Also, causalities remain unsolved, and multiple possible mechanisms of impact on the target species can be distributed along the gradients included to models. Apart from these limitations, the results improve our understanding on how *M*. *trossulus* responds to changes in the environment on a regional scale.

Our study confirms that environmental gradients largely differed in their ecological impact. Although it is expected that the magnitude of variability along a direct environmental gradient translates to the magnitude of environmental impacts on biota, the results showed otherwise. Specifically, our data spanned the full gradient of macroalgal cover and included all sediment types, the responses to these environmental variables were not very strong. On the other hand, salinity varied only between 4 and 8 but had a disproportionally large impact on *M*. *trossulus*. This suggests that species differ in their tolerance to different environmental factors, often showing lack of responses along a broad range of environmental variability.

According to Gleason [71] maximum abundance and limits of distribution of individual species are independently distributed along gradients. Indeed, our study clearly shows that direct environmental gradients have strong separate effects on the distribution of *M*. *trossulus*. This suggests that *M*. *trossulus* may inhabit a broad range of environmental conditions while avoiding very stressful extremes [7]. Instead, the probability of occurrence of species is skewed towards intermediate levels of environmental gradients i.e. within an optimum niche range [72,73]. Within these intermediate levels, however, direct environmental gradients are expected to highly interact with resource gradients, the latter determining the intensity of the biotic interactions such as competition. Although higher abundances are generally expected at high levels of the resource gradient, the values are often flattened by environmental stress at very high food concentrations [74]. Specifically, at high end of chlorophyll *a* gradient the biomass of mussels is unexpectedly low. This potentially refers to situations when high amount of particles in water column impair the feeding efficiency of mussels. Only at elevated exposure, when the accumulating organic matter is constantly resuspended and flushed away to deeper areas, mussels gain their biomass at high chlorophyll *a* values. Huston [74] also marks that fundamental niches are clustered at high levels of resource gradients. However, as optima of direct environmental and resource gradients do not spatially overlap, the realized optima fall at the highest resource levels still hospitable to the species [75].

Many intertidal communities are thought to be structured primarily by competitive interactions [75]. Subtidal communities characterized by sessile suspension feeders, however, have to cope with two limitations: the availability of substratum to which they attach to and the availability of food defined by processes in the pelagic ambient [64]. *Mytilus* is potentially capable of monopolizing the resources and completely excluding other species [75]. Yet, the combined effects of direct environmental and resource gradients likely prevent the monopolization of space by the sessile suspension feeder in the study area.

The importance of wave exposure has been shown to be one of the most significant physical factors locally influencing the distribution of mussels in the Baltic Sea [1,7,39]. In the previous studies, however, exposure was often considered as a direct mechanical disturbance to benthic populations. Specifically, the intense wave action renders habitat inhospitable for the attached organisms, has disturbing effect on mussel shells and puts them at risk of damage or dislodgement [50]. The decline in biomass at extreme exposure sites is likely due to negative effects of intense waves on larger mussels [7,45]. However, exposure may influence mussels also indirectly by affecting water temperature, oxygen level, substrate type and sedimentation rates [76–78]. For instance, positive indirect relationship has been suggested between wave exposure and mussel settlement resulting from the impact of exposure on accumulation of fine sediments [45].

However, our study suggests that, when decoupled from substrate type, temperature, and oxygen rates, exposure shows positive relationship with mussel distribution and biomass. Food supply is a crucial factor for benthic suspension feeders with sedentary lifestyle, and among environmental gradients analysed in our study, only exposure and water chlorophyll *a* reflect the food supply for mussels [9,45,50,79]. Mussels are able to deplete near-bottom water layer quickly from food [25] and will starve even with lush phytoplankton in water, if there is insufficient water movement. Thus, in large part, the separate effect of exposure most likely describes the exchange of phytoplankton biomass between coastal and open sea as well as between water surface and bottom layers, thereby indicating the intensity of phytoplankton flux into the site i.e. the availability of food resources in the area [80]. These results demonstrate the power of BRT analysis as traditional statistical analyses have trouble handling such multitude of interactions involving complex non-linearities.

Our analysis provides a strong numerical support for the earlier arguments that seabed structure is one of the most significant factor directly affecting local distribution of mussel populations [1,48,81,82]. The model indicates that the occurrence of mussels increases with elevated cover of stones. Both intertidal and subtidal environments are characterized by strong hydrodynamic forces. To remain in place, mussels have to be attached to substrate. Higher preference of mussels to hard bottoms may be related to sediment stability as gravel and pebbles can be more easily dislodged [83–86]. Moreover, sediment characteristics may indirectly relate to the availability of food as turbulence is expected to be higher over a rough solid substrate in comparison with a smooth substrate thereby facilitating the transport of chlorophyll *a* into near-bottom environments i.e. increasing food supply in the benthic boundary layer [25,47,87].

With increasing cover of silt and sand, the probability of occurrence of *M*. *trossulus* decreased. In addition to high instability of silt and sand, there are plausibly two other mechanisms involved. Firstly, high amount of silt particles in water column can impair the feeding efficiency of mussels [88,89] by damaging the filtering apparatus and disrupting the intake of food [90]. Secondly, a proximity to sandy substrate enhances mechanical abrasion under strong wave exposure [91].

Direct disturbance by ice often contributes to vertical distribution of mussels and other benthic organisms. Ice modifies sediment, tills seabed, crushes and dislodges benthic biota [92]. Depending on water depth, disturbance by ice can range from being very important to negligible [93]. In our study we used the temporally averaged values of ice thickness i.e. a proxy for ice impact on seascape and/or regional scale. Although, ice disturbance is expected to create high patchiness of the biota also at fine scales, this was not a focus of the current study. Our results show that the probability of occurrence of *M*. *trossulus* is the highest at intermediate ice scour. A likely explanation is that at shallow depths providing the plenitude of light and a lack of physical disturbance, macroalgae achieve complete dominance over *M*. *trossulus*. On the contrary, under moderate ice disturbance, *M*. *trossulus* recovers quicker than canopy-forming macroalgae and thereby gains a competitive advantage. However, if the intensity of ice impact is too strong, the populations of *M*. *trossulus* cannot recover and the occurrence of mussels is expected to decrease.

We found a strong interactive effect of exposure and ice on the probability of occurrence of *M*. *trossulus*. We may speculate that under low rates of ice disturbance the presence of *M*. *trossulus* is defined by e.g. substrate availability. With increasing ice disturbance and subsequent removal of *M*. *trossulus* individuals, however, the recovery is a function of the rates of water exchange. Exposure may act as a resource gradient with more intensive water exchange resulting in quicker growth of mussels [9,50,79]. Alternatively, elevated wave energy may dislocate living mussels from adjacent areas [50] and increase the probabilities of recolonization.

Plant cover is the only predictor variable included to the analysis that potentially displays biotic interactions with mussels. Algal cover has been identified as an important habitat factor for bivalves [94]. Mussels and plants as sessile organisms are competing for the same resources. In tidal areas, spatial competition between mussels and algae has been extensively studied and mussels are generally found to be superior competitors [95]. There can also be several facilitative interactions between mussels and macrophytes. Macroalgae increase complexity of the substrate and can function as attachment structures for the mussels at sites with high loads of sediments [7]. Besides, low intertidal and subtidal regions covered with filamentous algae, hydroids and bryozoans can offer blue mussels refuges from environmental stress and predation [85]. Algae create a heterogenic environment and can increase near-bottom flow turbulence and hence food availability [7]. In the study area, mussels have been observed to use algae as a substrate, which may offer the benefit of better access to food [25].

Our model indicates the lowest probability to find mussels at very sparsely vegetated or unvegetated bottoms, while mussel biomass pattern is modified by exposure: at exposed locations, mussel biomass tends to increase with the amount of vegetation, while at less exposed areas, the highest biomass occurs when plants are absent. These findings can be related to various types of interactions. For instance, at most exposed sites, wave disturbance may similarly detach both mussels and algae, thereby causing similar patterns of low biomass or absence, while at less exposed locations, spatial competition may play a role. Alternatively, mussels may provide extra nutrients for algae and thereby increase substantially their cover at higher exposure [52]. Contrary to tidal areas, our dataset suggests that mussels are not superior spatial competitors over macrophytes in the studied subtidal range. This is supported by the fact that the negative interaction occurs only at low exposure levels i.e. it seems unlikely that mussels at such a low abundance characteristic to low exposure sites have managed to gain a competitive advantage sufficient to exclude plants. A more possible explanation would be that as environmental conditions become too unfavourable to plants, mussels will gain more biomass. There may be another gradient hidden behind this pattern, like depth, which may suppress both plants and mussels albeit via different pathways, and modify the relationship between plants and mussels over the full extent of studied exposure gradient. It is obviously complicated to add biotic interactions to the niche model as these involve mutual interactions [96]; still, this seems fully accurate first step in understanding biotic patterns as the realized niche of almost any species inevitably includes biotic interactions [10].

Salinity defines the regional patterns of *M*. *trossulus* in the Baltic Sea range [42]. In the study area, where salinity may decrease down to 3 [97], *M*. *trossulus* live at the edge of their salinity tolerance [42]. Specifically, the lower salinity limit of *M*. *trossulus* is 4.5 in the study area [98,99]. Below this threshold, mainly due to high costs of osmoregulation, the mussel’s growth and reproduction become impossible [27,42,99]. Our model shows that above this threshold the biomass of *M*. *trossulus* increased sharply, and levelled off at salinities over 6. A linkage between salinity and the biomass of mussels may be also attributed to osmotic stress, as the size of *M*. *trossulus* depends on salinity [18,100]. In comparison, it might be interesting to test whether species with less pronounced stress-dependency of size (e.g. the barnacle *Balanus improvisus* in the Baltic Sea or *Mytilus* species in more saline ranges) would respond solely to resource and biotic interaction gradients in their biomass.

Average surface temperature and oxygen concentrations appeared to be weak predictors for mussel occurrence with the probability of occurrence increasing with raising temperature and oxygen concentrations. However, our data did not cover very high temperatures and very low oxygen values that are suboptimal for *M*. *trossulus*, therefore this result should be treated with caution. Temperature in general may have an influence on mussel performance [101,102], but in the observed relatively low temperature range, the limitation of occurrence might be observed only during periods of extremely high water temperatures [103]. The study area is well aerated, therefore, mussels were probably not experiencing oxygen limitation.

We admit that there are certain difficulties in retrieving the cause-effect insight to species ecology from the modelling of species distribution. In spite of this, BRT models can provide useful ecological insights. The machine learning process was able to identify some ecologically meaningful separate effects and interactions that could be validated in future experiments. Modelling separately both the occurrence and biomass distribution of mussels enables better identify processes responsible for mussel recruitment and production. Species distributions are often modelled using heavily reduced biological information like presence-absence or presence-only data [13]. Such reduced information has been used occasionally also to include biotic interactions to species distribution models [104]. Nevertheless, biotic interactions are expected to manifest in population sizes rather than the range of occurrence of species and population sizes are in turn expected to affect intensities of biotic interaction [105]. The used methodology also enables to identify the tipping points of various environmental variables where even slight alterations lead to dramatic changes either in the probability of occurrence or the biomass of mussels. Such tipping points can also be incorporated into experiments to define ecologically meaningful factor levels.

The BRT modelling also showed that when a single and easy to measure parameter as water depth was used to regress the presence and biomass of *M*. *trossulus* the models explained only 30−37% of variability in the patterns of mussels. Although depth is traditionally regarded amongst the most important parameters describing spatial pattern of mussels, its usage is neither justified in its predictive performance nor due to inherent difficulties to interpret the cause-effect relationships involved. As such the results advocates for a selection process of model environmental variables that is based on the theoretical assumptions of the species-environment relationship rather than the availability of ambiguous easy-to-be-used proxies from existing databases.

The current BRT model did not explain 35% of variability in the biomass of *M*. *trossulus*. This limitation is most likely related to the properties of mesoscale hydrophysical, ice and chlorophyll *a* models that do not take into account fine-scale variability in these key environmental variables. Mussel populations, however, are very patchy at fine spatial scales. Moreover, the current pattern of mussel distribution may be conditioned by rare stochastic recruitment and/or disturbance events that took place some decades ago but are not represented e.g. in the contemporary weather climate [106,107].

To conclude, our analyses suggest that distribution pattern of *M*. *trossulus* in the studied subtidal area is largely set by separate effects of direct environmental gradients whereas within its suitable habitat range, resource gradients have an important role in shaping the biomass distribution of *M*. *trossulus*. The developed BRT model appears to perform well compared to e.g. traditional spatial descriptive models but enables to describe the realized niche in detail while simultaneously explaining the variability in the stock size of the species. Further research may be targeted to understand the large scale patterns of *M*. *trossulus* over its full environmental range, as well as to test the generality of our results about the dependence of realized niches and population sizes on different types of environmental gradients across other organism groups.
